# Migraine incidence and coffee consumption among child-bearing age women: the Korea Nurses’ Health Study

**DOI:** 10.1038/s41598-024-53302-x

**Published:** 2024-06-04

**Authors:** Chiyoung Cha, Oksoo Kim, Yanghee Pang, Hyunseon Jeong, Jung Eun Lee, Heayoung Lee, Hyunju Dan

**Affiliations:** 1 College of Nursing, Ewha Womans University, Seoul, Korea; 2 System Health and Engineering Major in Graduate School, Ewha Research Institute of Nursing Science, Ewha Womans University, Seoul, Korea; 3 Department of Nursing, Seoil University, Seoul, Korea; 4 College of Nursing, Seoul Woman’s College of Nursing, Seoul, Korea; 5 Department of Food and Nutrition, College of Human Ecology, Seoul National University, Seoul, Korea; 6 Research Institute of Human Ecology, Seoul National University, Seoul, Korea; 7 Department of Nursing, Doowon Technical University, AnSung-Si, Korea; 8 Department of Nursing, Hwasung Medi-Science University, 400-5, Namyangchungang-Ro, Namyang-Eup, Hwasung-Si, 18274 Kyunggi-Do Korea

**Keywords:** Neurology, Risk factors

## Abstract

This longitudinal study aimed to identify factors that influence migraines in Korean women nurses using data from the Korea Nurses’ Health Study. Among those who participated in Survey 1 (2013–2014) and the follow-up survey (2015–2019), we selected 2605 participants for Cox proportional hazard regression analysis, 521 participants who were newly diagnosed with migraine and 2084 controls using a 1:4 incidence density sampling approach. Consuming coffee (≥ 3 cups: RR = 1.666; 95% CI = 1.175–2.362, < 3 cups: RR = 1.439; 95% CI = 1.053–1.966), being obese (BMI ≥ 25: RR = 1.341, 95% CI = 1.003–1.793), and engaging in vigorous physical activity (RR = 1.010; 95% CI = 1.000–1.019) increased the risk of developing a migraine. Nurses with an annual salary greater than $3500 were less likely to develop migraines (RR = 0.786, 95% CI = 0.631–0.979). The results imply that lifestyle factors, such as the amount of coffee consumption, BMI level, and degree of physical activity could be considered when formulating treatment plans for women who have newly developed migraines.

## Introduction

According to the 2018 Global Burden of Disease Study, the global prevalence of migraines has been gradually increasing, with East Asia reporting the highest increase during the past three decades^1^. The prevalence of migraine is 11%–20% and 3%–8% for males and females, respectively^2^. Hormonal changes that occur with menstruation have been suggested as a factor influencing migraines^3,4^. Migraines are reported to be a risk factor for cardiovascular disease^5^ and neurologic disorders^6^. However, as people suffering from migraines are unlikely to consult a physician or seek professional treatment, emphasizing migraine treatment and management could improve public health^7^.

Coffee, a caffeinated beverage, lowers the risk of cardiovascular disease, diabetes, and liver disease^8^; however, coffee is reported to induce symptoms in some migraine patients^9,10^. Recent studies found that coffee is an insignificant predisposing factor to migraine development^11,12^, and a randomized study showed no association between coffee consumption and the risk of migraine^11^. Thus, the influence of caffeine on human blood flow and arteries remains controversial^13^. In particular, since the effect of coffee consumption on health may vary by disease, guidelines on the effects of coffee on various diseases are needed.

Migraines are also influenced by other lifestyle factors. According to a systematic review, the research results on the association between migraine and circadian rhythm changes due to night shift work were inconsistent^14^. Therefore, the relationship between shift work exposure and the occurrence of migraine needs to be confirmed. Alcohol can trigger migraines^10^ and increase the symptoms of patients with migraines compared with those with non-migraine headaches^15^. The relationship between body mass index (BMI) and migraines has been continuously reported. Lucchesi et al.^16^ found that chronic migraine patients had a higher BMI score than episodic migraine patients, and a meta-analysis of primary headache influencing factors, including migraines, reported a correlation between high BMI and higher prevalence^17^. In particular, there was a significant relationship between obesity and migraine in reproductive aged women, but not in women aged 55 or older^18^. Therefore, it is necessary to confirm the relevance in this study targeting women of childbearing age.

Psychological health also influences migraines. Sleep deprivation and fatigue are common in patients with migraines^16,19^, and patients with chronic migraines have a higher degree of fatigue and lower sleep quality than patients with episodic migraines^16^. Patients with migraines often have mood disorders, such as anxiety, with approximately 43% of patients with migraines exhibiting anxiety symptoms^20^. In patients with migraines, mood disorders (e.g., anxiety and depression) can further increase disability, which may be reflected in missed days of work or school^21^. Therefore, it is necessary to investigate the effects of anxiety and depression on migraine development.

While many studies have focused on the onset of migraine symptoms, few have examined migraine incidence. In addition, there is insufficient research that identify factors affecting migraine development in females in East Asia who are at high risk for migraines. Therefore, in this study, the influence of lifestyle and psychological factors on the occurrence of migraine in females in Korea was investigated through longitudinal analysis.

## Materials and methods

### Study design, population, and participants

**Figure 1:**
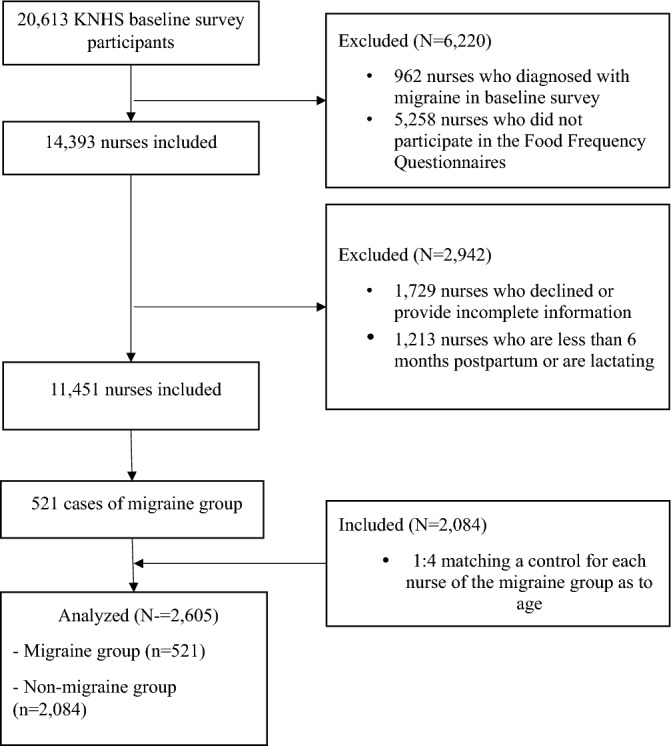
Study flow diagram.

We conducted a nested, 1:4 matched case–control study using data from the Korean Nurses' Health Study (KNHS) to investigate the risk factors for migraines. The KNHS is a cohort study that began in 2013 with 20,613 female nurses aged 18–45 as participants^22^. Eleven follow-up online surveys have been carried out since 2013. In this study, we used data collected between 2013 and 2014 as the baseline data. We excluded participants with a history of migraines, those who were breastfeeding or who had recently given birth, and those who did not complete the FFQ, which left us with 11,451 participants (see Fig. 1). Among those who participated in a follow-up survey between 2015 and 2019, we identified participants who were newly diagnosed with migraines (n = 521) and used the incidence density sampling approach^23^ to identify the matching group (n = 2,084). However, two participants in the matched group were excluded because they did not report their height. Thus, the total number of participants included in the BMI analysis was 2,603.

### Data collection and measures

Sociodemographic, lifestyle-related, and psychological variables were used to identify factors that affect migraine incidence. The sociodemographic factors were age, education, marital status, annual salary, and menstrual regularity. For menstrual regularity, participants were asked to report on a five-point Likert-type scale (1 = very regular, 2 = regular, 3 = irregular, 4 = very irregular, and 5 = amenorrhea), after which their responses were categorized as regular and irregular.

The lifestyle-related factors were shift work, night shift work, BMI, coffee consumption, alcohol consumption, physical activity, and vigorous physical activity. BMI was calculated based on the reported body weight and height. Participants reported their coffee consumption on the food frequency questionnaire (FFQ) and answered additional questions. Specifically, participants were asked how often they drank instant stick coffee, black coffee, café latte or cappuccino, and other kinds of coffee (e.g., café mocha) on average over the past year, and what amount they consumed each time. They selected one of ten responses for their coffee drinking frequency (rarely/less than once a month, 2–3 times a month, once a week, 2–4 times a week, 5–6 times a week, once a day, twice a day, 3 times a day, 4 times a day, and 5 or more times a day). In addition, participants estimated the amount of each drink via five responses (one very small cup (192 ml), one small cup (237 ml), one medium cup (355 ml), one large cup (437 ml), and very large cup (592 ml)). The total coffee intake per day (ml/day) was obtained by multiplying the number of times drunk per day for each type of coffee by the amount of coffee consumed per serving, and the total coffee intake per day (ml/day) was divided by the average amount per cup (i.e., 285 ml) to determine the coffee consumption (cup/day). Coffee consumption was categorized into not drinking, drinking less than three cups and three or more cups. Regarding alcohol consumption, participants reported how often they had drunk on average over the past year and how many drinks they consumed each time. For frequency of drinking, participants chose from among five responses: never drank, once a month, 2–4 times a month, 2–3 times a week, and 4 or more times a week). The amount of drink per serving was answered as a continuous variable. The number of times of drinking was converted to the frequency of intake per day and then multiplied by the amount of drinking per time to obtain the amount of drinking per day. Physical activity was measured by estimating activities throughout the previous year using the metabolic equivalent of task (MET), which is a measure of the metabolic rate ratio during exercise, with 1 MET equal to the amount of energy required to sit still^24^. The 11 physical activities included walking (for exercising or work), jogging, running (10 km/h or more), cycling (including bicycle exercise equipment), playing tennis (including squish and racquetball), round-trip swimming in a pool, other aerobic exercises (dancing, skiing, stepper, etc.), light exercise (yoga, stretching, calisthenics), other vigorous physical activities, weight training (arm exercises), and weight training (leg exercises). The average activity time per week for each physical activity was classified into the following ten responses: never, 1–4 min, 5–19 min, 20–59 min, 1 h, 1–1.5 h, 2–3 h, 4–6 h, 7–10 h, and over 11 h. Vigorous physical activity included seven activities (jogging, running, cycling, playing tennis, pool swimming, other aerobic exercise, and other vigorous physical activity) with a MET value of six or higher among the physical activities. For physical activity and vigorous physical activity, MET hours/week were obtained by multiplying the MET value for each physical activity after obtaining the representative value in the unit of time. A validated questionnaire was used for coffee consumption, alcohol consumption, physical activity, and vigorous physical activity, and experts reviewed the conversion process of each variable.

The psychological factors included sleep disturbance, fatigue, stress, anxiety, and depressive symptoms. Sleep disturbance was measured with the Jenkins Sleep Evaluation Questionnaire^25^, which is composed of four items and answered on a 6-point Likert scale (0 = never to 5 = 22-31 days). Scores range from 0 to 20, with values of > 12 indicating frequent sleep disturbance and scores between 2 and 11 indicating occasional disturbance. Cronbach's α for this scale in this study was 0.868. Fatigue was assessed with the Chalder Fatigue Scale^26^, which consists of 11 items answered on a 4-point Likert-type scale (0 = less than usual to 3 = much more than usual). The range of scores is 0–33, with higher scores indicating greater fatigue. In this study, the Cronbach’s α was 0.920. Stress was measured with the Perceived Stress Scale-10 (PSS-10)^27^, which consists of ten items answered on a 5-point Likert-type scale (0 = never to 4 = very often). The range of scores is 0–40, and higher scores indicate greater stress levels. In this study, Cronbach’s α of the PSS-10 was 0.568. Anxiety was measured using the Generalized Anxiety Disorder-7 (GAD-7) scale^28^, which consists of seven items answered on a 4-point Likert-type scale (0 = not at all to 3 = almost daily). The range of scores is 0–21, and higher scores indicate greater anxiety levels. In this study, Cronbach’s α of GAD-7 was 0.684. Depressive symptoms were measured using the Patient Health Questionnaire^29^, which consists of nine items answered on a 4-point Likert-type rating (0 = not at all to 3 = almost daily). The range of scores is 0 to 27, and higher scores indicate greater depressive symptoms. In this study, Cronbach’s α was 0.895.

The incidence of migraines was measured using a one-item question: Have you ever been diagnosed with a migraine, and if so, in what year was it diagnosed?

### Data analysis

The data from this study were analyzed in SPSS® version 26. Differences between the group with a newly diagnosed migraine and the group without a migraine were analyzed using chi-square tests, t-tests, and the Mann–Whitney U test. To analyze factors that influenced the incidence of migraine as time-varying explainable variables, the relative risks were calculated in a Cox proportional hazards regression analysis^30^.

### Ethical considerations

This study was reviewed and approved by the Institutional Review Board at the principal investigator’s institution (Approval No. 2013-03CON-03-P, 117-4, ewha-201904-0012-01). The study participants were able to review the research purpose, voluntariness, and confidentiality before signing the informed consent form and participating in the online survey. Collected data were de-identified and stored in a secure place accessible to the principle investigator and data managers.

## Results

### Comparisons between the newly diagnosed migraine group and non-migraine group

Table 1 describes the differences between the group with newly diagnosed migraines and the group without migraines. Among the sociodemographic factors, the group newly diagnosed with migraines contained more people with menstrual irregularity (29.0%) than their counterparts without migraines (23.4%) (χ^2^ = 6.976; *p* = 0.008). Likewise, the group newly diagnosed with migraines had higher scores for sleep disturbance (*t* = − 4.127; *p* < 0.001), fatigue (*t* = − 4.244; *p* < 0.001), stress (*t* = − 3.515; *p* < 0.001), anxiety (*t* = − 4.568; *p* < 0.001), and depressive symptoms (*t* = − 4.188; *p* < 0.001) than the comparison group.

**Table 1:** General characteristics of newly diagnosed migraine and non-migraine group (N = 2605).

Variables	Total	Migraine		*t* or* X*^*2*^* or U/Z*	*p*
		Yes (n = 521)	No (n = 2084)		
	N (%) or M ± SD	N (%) or M ± SD or Mean rank(M ± SD)	N (%) or M ± SD or Mean rank(M ± SD)		
Sociodemographic factors					
Age					
≤ 29	1668 (64.0)	337 (64.7)	1331 (63.9)	0.155	0.925
30–39	733 (28.1)	143 (27.4)	590 (28.3)		
≥ 40	204 (7.8)	41 (7.9)	163 (7.8)		
Level of education					
3-year college	1318 (50.6)	252 (48.4)	1066 (51.2)	1.712	0.425
4-year university	1104 (42.4)	234 (44.9)	870 (41.7)		
Master or higher	183 (7.0)	35 (6.7)	148 (7.1)		
Marital status					
Married	786 (30.2)	155 (29.8)	631 (30.3)	0.055	0.831
Single/others	1819 (69.8)	366 (70.2)	1453 (69.7)		
Annual salary ($)					
≤ 3500	1752 (67.3)	368 (70.6)	1384 (66.4)	3.375	0.066
> 3500	853 (32.7)	153 (29.4)	700 (33.6)		
Regularity of menstruation					
Regular	1966 (75.5)	370 (71.0)	1596 (76.6)	6.976	0.008
Irregular	639 (24.5)	151 (29.0)	488 (23.4)		
Lifestyle factors					
Shift work					
Yes	1963 (75.4)	381 (73.1)	1582 (75.9)	1.738	0.187
No	642 (24.6)	140 (26.9)	502 (24.1)		
Night shift work					
Yes	1809 (69.4)	352 (67.6)	1457 (69.9)	1.086	0.297
No	796 (30.6)	169 (32.4)	627 (30.1)		
BMI					
Underweight (< 18.5)	405 (15.6)	76 (14.6)	329 (15.8)	7.720	0.052
Normal (18.5–22.9)	1753 (67.3)	343 (65.8)	1410 (67.7)		
Overweight (23–24.9)	245 (9.4)	47 (9.0)	198 (9.5)		
Obese (≥ 25)	200 (7.7)	55 (10.6)	145 (7.0)		
Coffee consumption (cup/day)					
None	284 (10.9)	45 (8.6)	239 (11.5)	5.573	0.062
< 3	1784 (68.5)	354 (67.9)	1430 (68.6)		
≥ 3	537 (20.6)	122 (23.4)	415 (19.9)		
Alcohol consumption (drink/day)*		1303.4	1302.9	542,674.0/− .014	0.989
		(0.17 ± .309)	(0.20 ± .381)		
Physical activity (MET-hours/week)*		1268.48	1311.63	524,895.0/− 1.172	0.241
		(21.88 ± 37.470)	(21.50 ± 31.437)		
Vigorous physical activity (MET-hours/week)*		1274.84	1310.04	528,209.0/− .992	0.321
		(8.52 ± 26.401)	(7.41 ± 19.386)		
Psychological factors					
Sleep disturbance	6.84 ± 5.135	7.69 ± 5.363	6.62 ± 5.055	− 4.127	< .001
Fatigue	17.89 ± 6.198	18.92 ± 6.078	17.63 ± 6.202	− 4.244	< .001
Stress	6.50 ± 2.352	6.83 ± 2.340	6.42 ± 2.349	− 3.515	< .001
Anxiety	50.68 ± 10.090	52.48 ± 10.878	50.23 ± 9.834	− 4.568	< .001
Depressive symptoms	7.45 ± 5.635	8.43 ± 6.083	7.27 ± 5.488	− 4.188	< .001

*Mann–Whitney tests were conducted in violation of the assumption of normality.

### Comparisons according to coffee consumption

Table 2 presents the differences among the group who did not drink coffee, the group who drinks < 3 cups a day, and the group who drinks coffee ≥ 3 cups a day. Among the sociodemographic factors, the group that drank three or more cups of coffee had a higher percentage of participants aged 40 or older (*χ*^*2*^ = 102.925, *p* < 0.001), with a master’s degree or higher (*χ*^*2*^ = 25.048, *p* < 0.001), married (*χ*^*2*^ = 46.105, *p* < 0.001), with an annual income of more than $3,500 (*χ*^*2*^ = 28.069, *p* < 0.001), and who menstruated regularly (*χ*^*2*^ = 6.212, *p* = 0.45) compared to the comparison group. Among the lifestyle factors, the group who drank ≥ 3 cups of coffee contained more participants who did not do shift work (*χ*^*2*^ = 13.739, *p* = 0.001) and night shift work (*χ*^*2*^ = 11.387, *p* = 0.003), and were overweight or obese (*χ*^*2*^ = 24.384, *p* < 0.001) than the group who drank < 3 cups of coffee or who did not drink coffee.

**Table 2:** General characteristics according to coffee consumption (N = 2,603).

Variables	Coffee consumption (cup/day)			*F* or* X*^*2*^	*p*
	None	< 3	≥ 3		
	N (%) or M ± SD	N (%) or M ± SD or Mean rank	N (%) or M ± SD or Mean rank		
**Sociodemographic factors**					
Age					
≤ 29	207 (72.9)	1210 (67.8)	251 (46.7)	102.925	< .001
30–39	65 (22.9)	462 (25.9)	206 (38.4)		
≥ 40	12 (4.2)	112 (6.3)	80 (14.9)		
Level of education					
3-year college	157 (55.3)	916 (51.3)	245 (45.6)	25.048	< .001
4-year university	109 (38.4)	765 (42.9)	230 (42.8)		
Master or higher	18 (6.3)	103 (5.8)	62 (11.5)		
Marital status					
Married	67 (23.6)	494 (27.7)	225 (41.9)	46.105	< .001
Single/others	217 (76.4)	1290 (72.3)	312 (58.1)		
Annual salary ($)					
≤ 3500	218 (76.8)	1216 (68.2)	318 (59.2)	28.069	< .001
> 3500	66 (23.2)	568 (31.8)	219 (40.8)		
Regularity of menstruation					
Regular	204 (71.8)	1337 (74.9)	425 (79.1)	6.212	.045
Irregular	80 (28.2)	447 (25.1)	112 (20.9)		
Lifestyle factors					
Shift work					
Yes	215 (75.7)	1376 (77.1)	372 (69.3)	13.739	.001
No	69 (24.3)	408 (22.9)	165 (30.7)		
Night shift work					
Yes	199 (70.1)	1269 (71.1)	341 (63.5)	11.387	.003
No	85 (29.9)	515 (28.9)	196 (36.5)		
BMI					
Underweight (< 18.5)	56 (19.9)	285 (16.0)	64 (11.9)	24.384	< .001
Normal (18.5–22.9)	182 (64.5)	1219 (68.3)	352 (65.5)		
Overweight (23–24.9)	23 (8.2)	163 (9.1)	59 (11.0)		
Obese (≥ 25)	21 (7.4)	117 (6.6)	62 (11.5)		
Alcohol consumption (drink/day)^§^	1147.65	1318.56	1333.45	14.929	.001
Physical activity (MET-hours/week)^§^	1247.80	1310.67	1306.71	1.729	.421
Vigorous physical activity (MET-hours/week)^§^	1261.39	1310.30	1300.75	1.122	.571
Psychological factors					
Sleep disturbance	7.15 ± 5.371	6.81 ± 5.029	6.76 ± 5.356	.630	.533
Fatigue	18.00 ± 6.643	17.90 ± 6.139	17.78 ± 6.159	.132	.876
Stress	6.75 ± 2.427	6.51 ± 2.316	6.35 ± 2.423	2.730	.065
Anxiety	51.54 ± 10.505	50.71 ± 9.987	50.11 ± 10.187	1.906	.149
Depressive symptoms	7.87 ± 6.103	7.43 ± 5.527	7.30 ± 5.735	1.005	.366

^§^Kruskal–Wallis tests were conducted in violation of the assumption of normality.

### Factors that influence migraine incidence

Table 3 describes the Cox proportional hazards regression analysis for the incidence of migraines. Among the variables, coffee consumption had the greatest relative risk for the incidence of migraines, followed by BMI level, annual salary level, and vigorous physical activity level, when other variables were controlled. Those who had ≥ 3 cups of coffee (RR = 1.666; 95% confidence interval (CI) = 1.175–2.362) and those who had < 3 cups of coffee (RR = 1.439; 95% CI = 1.053–1.966) had a higher risk of developing migraines than those who did not drink coffee. Those whose BMIs were ≥ 25 (RR = 1.341; 95% CI = 1.003–1.793) had a higher relative risk of developing migraines compared to those who had BMIs between 18.5 and 22.9. Those with an annual salary greater than $3,500 were less likely than their counterparts to develop migraines (RR = 0.786, 95% CI = 0.631–0.979). Those who engaged in vigorous physical activity had a slightly higher relative risk of developing migraines than their counterparts (RR = 1.010; 95% CI = 1.000–1.019).

**Table 3:** Relative risk (RR) of migraine incidence (N = 2,603).

Variables	HR	95% CI for hazard ratio		*p*
Sociodemographic factors				
Age				
≤ 29	Ref			
30–39	.950	.730	1.236	.702
≥ 40	.906	.593	1.384	.647
Level of education				
3-year college	Ref			
4-year university	1.046	.868	1.260	.638
Master or higher	1.062	.716	1.576	.765
Marital status				
Single/others	Ref			
Married	1.058	.817	1.371	.669
Annual salary ($)				
≤ 3500	Ref			
> 3500	.786	.631	.979	.032
Menstruation				
Regular	Ref			
Irregular	1.174	.966	1.427	.108
Lifestyle factors				
Shift work				
No	Ref			
Yes	.830	.554	1.245	.368
Night shift work				
No	Ref			
Yes	1.004	.678	1.488	.983
BMI				
Normal (18.5–22.9)	Ref			
Underweight (< 18.5)	1.024	.796	1.318	.851
Overweight (23–24.9)	1.040	.764	1.415	.804
Obese (≥ 25)	1.341	1.003	1.793	.048
Coffee consumption (drink/day)				
None	Ref			
< 3	1.439	1.053	1.966	.022
≥ 3	1.666	1.175	2.362	.004
Alcohol consumption	.855	.657	1.112	.243
Physical activity	.995	.989	1.001	.108
Vigorous physical activity	1.010	1.000	1.019	.039
Psychological factors				
Sleep disturbance	1.017	.996	1.038	.112
Fatigue	1.004	.985	1.023	.701
Stress	1.009	.964	1.055	.711
Anxiety	1.011	.999	1.022	.066
Depressive symptoms	1.005	.982	1.028	.688

All the variables listed above were adjusted in the Cox proportional hazards model.

## Discussion

In this study, we examined the variables that influence the incidence of migraines among female nurses using KNHS data.

To understand our data, we compared the differences between those who were newly diagnosed with migraine and those without migraines. The group who was newly diagnosed with migraines had more women who were experiencing menstrual irregularities than did the other group. This difference is understandable in that the incidence of migraine is influenced by hormonal changes, particularly estrogen^31^, and an irregular menstrual cycle is frequently cited as a key trigger for migraine attacks^32,33^. To develop an integrative healthcare strategy, females who have just been diagnosed with migraines should have their menstrual cycles evaluated. Additionally, the group with newly diagnosed migraines scored higher than the control group for fatigue and sleep disturbance. Similarly, a review article claims that fatigue and disturbed sleep are common symptoms of migraines and may have a biological basis^34^. In a Korean study that examined migraine diaries, 55.1% and 48.5% of those with sleep disturbances and fatigue, respectively, experienced migraine attacks^15^. Nurses frequently work night shifts and are overworked, which can cause fatigue and interrupted sleep. Therefore, strategies for managing sleep disturbance and fatigue might help to reduce the prevalence of migraine among nurses.

The group with newly diagnosed migraines exhibited greater stress, anxiety, and depression symptom scores than did the comparison group. Stress has long been known to be one of the main factors contributing to the occurrence of migraines^15,35^, and migraine sufferers are said to experience greater levels of stress than those without migraines^36^. Anxiety and depression symptoms have also been identified as migraine risk factors^37,38^. Evidence suggests that psychological comorbidities (e.g., anxiety and depression) are more common in migraine patients than in the general population^21,39,40^. Nurses often experience stress, worry, and despair, which can increase their risk of migraines^41^. Thus, nurses who experience migraines might need to have their psychological symptoms evaluated.

Next, we compared the differences in groups according to their coffee consumption. It is interesting to note that the group who drank ≥ 3 cups of coffee a day had more women with regular menstrual cycles, a BMI ≥ 25, and greater alcohol consumption but fewer women who worked shifts or night shifts. Previous studies^42,43^ found that caffeine consumption influences the menstrual cycle, while we reported more women with regular cycles in a group who drank ≥ three cups of coffee a day. Given this discrepancy, further studies are needed to explore the relationships between coffee consumption and menstrual cycle. The greater incidence of women with a higher BMI in the group who drank ≥ 3 cups of coffee more a day is in line with the finding of previous studies that obesity is positively related to a high intake of coffee^44^. In addition, although fewer women in the group who drank ≥ 3 cups of coffee a day worked shifts and night shifts in our study, Lawson et al.^45^ reported that people drank more coffee when working shifts, with the amount of coffee consumption increasing for night shifts^46^. The group who drank ≥ 3 cups of coffee a day had greater alcohol consumption than the other two groups. In a study conducted with Korean, US, and Italian nurses, a high proportion of Korean nurses reported drinking alcohol with highly caffeinated drinks compared to their counterparts in the other countries^47^.

Last, we conducted a Cox regression analysis to identify influencing factors for migraine incidence from a longitudinal perspective. Coffee consumption was the most influential factor in increasing migraine incidence, followed by BMI level, annual salary level, and vigorous physical activity level. Those who consumed ≥ 3 cups of coffee a day and < 3 cups a day were at 1.666- and 1.439-times higher risk of developing migraines, respectively, than those who did not drink coffee. We would like to highlight the predictive role of coffee consumption for migraine incidence from a longitudinal analysis in that although there have been conflicting results between these two variables, most evidence was drawn from cross-sectional studies^48,49^. Similarly, studies that analyzed diaries written for certain periods reported positive relationships between caffeinated drinks and migraine incidence^50,51^. Although coffee was examined in this study, previous studies investigated all caffeinated drinks^52^. Thus, more longitudinal studies are needed to explore the amounts of coffee and caffeine consumed and their contributions to migraine incidence.

Those who were obese (BMI ≥ 25) were at 1.341 times higher risk of developing migraines than those having a normal range BMI. Pavlovic et al.^18^ reported that obesity and migraine were positively related among childbearing-age women. In addition, a study of Chinese adults also found that migraine patients are more likely to be overweight or obese^53^. Therefore, assessment and prevention of migraine should be included in health plans for obese patients and the risk of migraine should be included in public campaigns to increase public awareness of the need to reduce obesity levels.

The risk of acquiring a migraine increased by 1.010 times for every additional MET hour of intense physical exercise per week. Conflicting evidence links vigorous exercise with migraines. According to a review study that examined the precipitating factors of migraines, 25% of migraine sufferers said that exercise caused their migraines^54^. On the other hand, another review study found that exercising reduced migraine frequency, severity, and duration^55^. To determine the connection between physical activity and migraine, more research is required.

Estrogen is strongly related to migraines in women^56^. Thus, it is worth noting that menstrual regularity was not a predicting factor for migraine incidence from a longitudinal analysis, and that a greater proportion of women reported regularly menstruating in the migraine group compared to their non-migraine counterparts. In line with our findings, a recent case–control study in Iran revealed that menstrual regularity is not significantly related to migraines while other menstrual characteristics, including period duration, oligomenorrhea, and polymenorrhea, are^57^.

Surprisingly, psychological factors (e.g., sleep disturbance, fatigue, stress, anxiety, and depressive symptoms) were not predicting factors for migraine incidence despite the fact that their scores were significantly higher for the migraine group than their non-migraine counterparts. Although numerous studies have provided evidence of the relationships between psychological factors and migraines, little analysis has been conducted from a longitudinal perspective. Our results reveal that psychological factors might be present with migraines but not play a significant role in predicting the incidence of migraines.

This study is noteworthy in that we attempted to explore the relationships between coffee consumption and migraine incidence using both group comparison and a longitudinal analysis. Nonetheless, it has certain limitations. The KNHS included nurses who worked in hospitals, making it difficult to generalize these findings. In addition, we excluded women who recently gave birth or were breastfeeding, which might have made us miss some incidences of migraines. Since we measured coffee consumption in cups, the caffeine content is unclear. Finally, neither the type nor the severity of migraines was evaluated.

## Conclusion

In a Cox regression analysis, coffee consumption contributed most to the incidence of migraines among female nurses, followed by being obese, having a higher annual salary level, and engaging in vigorous physical activities. Lifestyle-related factors, such as coffee consumption and vigorous exercise habits, should be evaluated and incorporated into the treatment plan for new-onset migraines among female patients.

## Data Availability

The data set are not publicly available. This is government data and it will take time to be able to clean the data and establish guidelines. Korea Disease Control and Prevention Agency plans to release the data to the public in the future.
